# Endothelin-1 and Norepinephrine Overflow from Cardiac Sympathetic Nerve Endings in Myocardial Ischemia

**DOI:** 10.1155/2012/789071

**Published:** 2012-06-25

**Authors:** Masashi Tawa, Satoshi Yamamoto, Mamoru Ohkita, Yasuo Matsumura

**Affiliations:** ^1^Laboratory of Pathological and Molecular Pharmacology, Osaka University of Pharmaceutical Sciences, Takatsuki, Osaka 569-1094, Japan; ^2^Department of Pharmacology, Shiga University of Medical Science, Otsu, Shiga 520-2192, Japan; ^3^Department of Pharmacy, Osaka Rosai Hospital, Sakai, Osaka 591-8025, Japan

## Abstract

In protracted myocardial ischemia, sympathetic activation with carrier-mediated excessive norepinephrine (NE) release from its nerve endings due to reversal of NE transporter in an outward direction is a prominent cause of arrhythmias and cardiac dysfunction. Endothelin-1 (ET-1) and its receptors are intimately involved in the regulation of this carrier-mediated NE overflow in protracted myocardial ischemia. The ET-1 system is often complex, sometimes involving opposing actions depending on which receptor subtype is activated, which cells are affected, and whether stimuli are endogenously generated or exogenously applied. Therefore, a detailed understanding of the ET-1 system is important for applying drugs acting on this system in clinical settings for the treatment of ischemic cardiac disease. This article provides a detailed analysis of how the ET-1 system is involved in the regulation of carrier-mediated NE release from sympathetic nerve endings in protracted myocardial ischemia.

## 1. Introduction

Enhancement of cardiac sympathetic nerve activity in myocardial ischemia elicits norepinephrine (NE) release from its nerve endings. Activation of *β*-adrenoceptors by NE causes positive inotropic and chronotropic effects which augment oxygen demand. By activating *α*-adrenoceptors, NE also provokes coronary vasoconstriction, which diminishes oxygen supply. The detailed mechanisms by which NE regulates cardiac contractility or coronary vasomotion have been well described in previous reports [1–6]. Both of these actions of NE are considered to be important factors that exacerbate myocardial ischemia. Indeed, animal and clinical studies have demonstrated that negative modulation of NE release, or blockade of its effects, significantly suppresses postischemic cardiac dysfunction and arrhythmias [7–11]. The mechanisms by which ischemia enhances cardiac sympathetic nerve activity and increases NE overflow have been discussed [7]. In cardiac sympathetic nerve endings in a protracted ischemic condition, free axoplasmic NE and H^+^ accumulate massively owing to the lack of a driving force for NE storage by ATP depletion. Increased axoplasmic H^+^ activates the Na^+^/H^+^ exchanger (NHE), which consequently leads to an influx of Na^+^ in exchange for H^+^. Furthermore, inhibition of Na^+^/K^+^ ATPase activity due to ATP depletion results in the accumulation of axoplasmic Na^+^. This Na^+^ accumulation triggers excessive axoplasmic NE release via reversal of the NE transporter (NET) from the intracellular to extracellular space [6, 12]. This carrier-mediated NE release in protracted myocardial ischemia depends on Na^+^ entry via NHE activation and is supported by evidence that NHE inhibitors are as effective as NET inhibitors in reducing NE overflow [9]. Although several physiologically active substances (e.g., histamine, adenosine, bradykinin, and angiotensin) contribute to the modulation of this release via respective receptors [9–11], endothelin-1 (ET-1) is also one of them. ET-1 was originally found as a 21-amino acid vasoconstrictor peptide produced by vascular endothelial cells [13]. Accumulating evidence indicates the crucial role of ET-1 in the pathogenesis and/or development of several cardiovascular diseases [14]. It has been reported that an ischemic event results in increases in ET-1 levels [15–18] and its binding sites [19, 20]. Additionally, blockade of its effects has been shown to act protectively against postischemic cardiac and/or coronary dysfunction both in animal studies [18, 21–29] and human clinical trials [30, 31]. These findings suggest that ET-1 plays an important role in the pathophysiology of myocardial ischemia. In the following paragraphs, we will summarize the interaction between the ET-1 system and NE overflow from cardiac sympathetic nerve endings in protracted myocardial ischemia.

## 2. ET-1 System in NE Overflow

ET-1 is generated from an intermediate form termed big ET-1 via proteolytic cleavage by endothelin-converting enzyme (ECE) [13]. The prime sites of ET-1 production in the heart are coronary and endocardial endothelial cells [32–34]. This peptide is well known to have several cardiovascular actions through the activation of its receptors, endothelin type A receptors (ET_A_Rs) and type B receptors (ET_B_Rs) [35]. Briefly, both receptor subtypes belong to the seven-transmembrane domain rhodopsin receptor superfamily, and signaling transduction of these receptors is coupled to multiple G-proteins (Gq, Gi/o, and Gs) [35]. These ET-1 receptors are widely distributed in the mammalian heart, such as the coronary vasculature [36], myocardium [37], and cardiac sympathetic nerve terminal [24].

### 2.1. ET_A_Rs in NE Overflow

Our previous study using the Langendorff technique indicated for the first time that ET_A_Rs modulate carrier-mediated NE release from cardiac sympathetic nerve endings [23]. In this report, we demonstrated that a selective ET_A_R antagonist ABT-627 markedly decreased NE overflow in coronary effluent after 40-min global ischemia and reperfusion in rat hearts. A similar result was reported by Isaka and associates [24], who found that ET_A_Rs blockade with BQ-123, a selective ET_A_R antagonist, significantly attenuated NE overflow during reperfusion after 20-min global ischemia in isolated perfused guinea pig hearts. In addition, they provided a definitive demonstration that ET_A_Rs exist in cardiac sympathetic nerve terminals of guinea pig hearts. These findings suggest that activation of ET_A_Rs existing in cardiac sympathetic nerve endings modulates carrier-mediated NE release in a stimulatory manner. As support for this proposal, we have reported that 40-min global ischemia-induced NE overflow was more highly observed in isolated perfused hearts of ET_B_R-deficient homozygous (*sl*/*sl*) rats than in hearts of wild-type rats, and an exaggerated response to prolonged ischemia in *sl*/*sl* rats was abolished by ABT-627 treatment, indicating that ET_A_R-mediated action is responsible for augmented NE overflow in *sl*/*sl*   rats [23].

The signaling mechanism of ET_A_R in stimulatory modulation of carrier-mediated NE release has been suggested. As mentioned above, NHE is an important regulator for carrier-mediated NE release from sympathetic nerve endings in protracted myocardial ischemia [9]. This transporter activity is well known to be regulated by a variety of G-protein coupled receptor (GPCR) systems [38]. In fact, several researchers have clarified the positive functional coupling of ET-1/ET_A_R and NHE at a cellular level [39, 40]. Consistent with this view, in isolated perfused hearts, pharmacological NHE inhibition by 5-(N-ethyl-N-isopropyl)-amiloride (EIPA) has been demonstrated to decrease excessive NE release induced by ET_A_Rs activation [23, 24]. Therefore, it is reasonable to consider that ET_A_Rs stimulation activates the NHE system at the level of cardiac sympathetic nerves. On the other hand, the second messengers mediating this response remain unclear. Because phospholipase C (PLC)-protein kinase C (PKC) cascade is known to activate the NHE system [38], the stimulatory action of ET_A_R on carrier-mediated NE release may result from an increase in PLC and/or PKC activity. However, Horinouchi and colleagues recently demonstrated that there are multiple intracellular signal transduction pathways for ET_A_R to activate NHE [40]. Briefly, they provided evidence for the existence of an NHE activating pathway mediated through p38 mitogen-activated protein kinase (p38 MAPK), not through PLC, in Chinese hamster ovary cells. Unfortunately, at present, we cannot assert which pathway plays a critical role in the positive regulation of NHE activity via ET_A_R in cardiac sympathetic nerves. It is hoped that the detailed mechanisms of how ET_A_R stimulates the NHE system and, thus, carrier-mediated NE release in protracted myocardial ischemia will be clarified.

In the above-mentioned previous studies using isolated perfused hearts, NE overflow reflected the severity of cardiac dysfunction after reperfusion. For example, ABT-627 improved left ventricular systolic and diastolic function after myocardial ischemia/reperfusion in rat hearts [23]. Additionally, BQ-123 completely diminished the incidence of ventricular fibrillation after global ischemia in guinea pig hearts [24]. Furthermore, more severe left ventricular dysfunction after myocardial ischemia/reperfusion was observed in *sl*/*sl*  rats than that in wild-type rats, and this severity in *sl*/*sl*  rats decreased by treatment with ABT-627 [23]. Basically, ET_A_R-mediated NE overflow is considered to contribute, at least in part, to ischemia/reperfusion-induced cardiac dysfunction.

### 2.2. ET_B_Rs in NE Overflow

It is still unclear whether or not ET_B_R directly interacts with carrier-mediated NE release. Our group and another group have confirmed that pharmacological blockade of ET_B_Rs by A-192621 (selective ET_B_R antagonist) and BQ-788 (selective ET_B_R antagonist), respectively, exaggerates NE overflow induced by protracted global myocardial ischemia in isolated rodent hearts [23, 24]. As mentioned in Section 2.1, we noted that 40-min global ischemia-induced NE overflow in isolated perfused hearts of ET_B_R-deficient *sl*/*sl*  rats was more highly observed than in hearts of wild-type rats. These responses to pharmacological blockade and the effects of genetic ET_B_Rs deficiency were almost completely abolished by ABT-627 treatment, indicating the possibility that ET_B_R itself does not play an important role in carrier-mediated NE release in ischemic hearts [23]. On the other hand, another group has noted that treatment with the selective ET_B_R agonist sarafotoxin S6c suppresses NE overflow during reperfusion after 20-min global ischemia in isolated perfused guinea pig hearts [24]. More recently, we have demonstrated that ET_B_Rs stimulation resulting from treatment with big ET-1 (see Section 2.5 for more details) suppresses NE overflow induced by 40-min global ischemia in isolated perfused rat hearts [41]. In contrast to the above idea, these findings indicate the possibility that ET_B_R negatively modulates carrier-mediated NE release from cardiac sympathetic nerve endings. The reason for this discrepancy may be due to differences between endogenous and exogenous stimuli. Briefly, the ability of ET_B_Rs stimulation by endogenously generated ET-1 may be extremely low. As such, the role of ET_B_R on carrier-mediated NE release from cardiac sympathetic nerve endings during protracted ischemia is not completely clear and further study is required.

Although it is unclear whether or not there is a direct contribution of ET_B_R to carrier-mediated NE release during myocardial ischemia, the amount of NE released from ischemic hearts has reflected cardiac dysfunction in studies introduced in this section. Briefly, treatment with A-192621 worsened left ventricular (both systolic and diastolic) function during reperfusion after 40-min global ischemia, and this detrimental effect of A-192621 was completely abolished by concomitant treatment with ABT-627 in rat hearts [23]. In addition, S6c prevented reperfusion arrhythmias (both incidence and duration of ventricular fibrillation) in guinea pig hearts [24]. On the other hand, BQ-788 did not alter the duration of ventricular fibrillation after 20-min global ischemia in guinea pig hearts [24].

### 2.3. Endogenously Generated ET-1 in NE Overflow

As described above, we have demonstrated that treatment with ABT-627 with or without the combination of A-192621 markedly suppressed NE overflow induced by protracted global myocardial ischemia to the same level in isolated perfused rat hearts using the Langendorff technique [23]. Isaka et al. also showed that the nonselective ET_A_R/ET_B_R antagonist PD-142893 significantly attenuated carrier-mediated NE release during 20-min global ischemia in isolated perfused guinea pig hearts [24]. These findings suggest that endogenous ET-1 contributes to carrier-mediated NE release by exclusively stimulating ET_A_Rs in ischemic hearts. This proposal was supported by our recent study which investigated the effects of ECE inhibition [18]. In this study using Langendorff perfused rat hearts, we observed that the selective ECE inhibitor SM-19712 suppressed excessive NE release from sympathetic nerve endings during 40-min global ischemia.

In the previous study, we showed that cardiac ET-1 levels were reduced during ischemia in rat hearts [18]. However, this does not mean that ET-1-induced action is diminished during myocardial ischemia. This is because ET-1 levels in coronary effluent from postischemic hearts were slightly, but not significantly, increased in our subsequent research (unpublished data). In fact, the magnitude of increase in ET-1 release observed during myocardial ischemia has been well documented [15, 16]. Although it is unclear whether or not ET-1 synthesis is increased in ischemic hearts from our research results, there is almost no doubt that the release of this peptide is increased. Released ET-1 during protracted myocardial ischemia may play an important role in carrier-mediated NE release via the activation of ET_A_Rs. Unfortunately, we did not check the effect of SM-19712 on ET-1 levels in coronary effluent from postischemic hearts. However, we have confirmed that cardiac ET-1 levels are not different before and immediately after prolonged ischemia in the case of SM-19712 treatment [18], suggesting the possibility that ET-1 release during myocardial ischemia is reduced by ECE inhibition. Taken together, endogenous ET-1 positively regulates protracted ischemia-induced NE overflow via the activation of ET_A_Rs, although it is unclear whether or not ET-1 is newly synthesized during ischemia.

Regarding cardiac function, PD-142893 and SM-19712 respectively shortened the duration of ventricular fibrillation [24] and improved left ventricular function [18] during reperfusion after myocardial ischemia relative to NE overflow. Therefore, endogenously generated ET-1 seems to be able to promote postischemic NE overflow, and this contributes, at least in part, to subsequent cardiac dysfunction.

### 2.4. Exogenously Applied ET-1 in NE Overflow

Exogenously applied ET-1 induces the same effects as endogenous ET-1 on carrier-mediated NE release from cardiac sympathetic nerve endings in ischemic hearts. In our study using isolated perfused rat hearts, exogenous ET-1 (0.03 and 0.1 nM) caused a marked and dose-dependent increase in NE overflow induced by 40-min global myocardial ischemia. Moreover, this action of exogenous ET-1 (0.1 nM) on NE overflow was completely suppressed by treatment with ABT-627 or EIPA [23]. In accordance with our findings, Isaka and associates also obtained results indicating that carrier-mediated NE release induced by 20-min global ischemia was augmented by exogenous ET-1 (0.1 and 1 nM) in a dose-dependent manner and this augmentation was counteracted with the combination of EIPA in isolated perfused guinea pig hearts [24]. Collectively, it is suggested that exogenously applied ET-1 positively regulates carrier-mediated NE release, possibly activating the NHE system through ET_A_Rs stimulation in protracted myocardial ischemia.

Exogenously applied ET-1 worsened cardiac dysfunction during reperfusion after global ischemia in association with the amount of NE released from ischemic hearts. In our study using isolated perfused rat hearts, exogenous ET-1 produced severe left ventricular systolic and diastolic dysfunction after ischemia/reperfusion in a dose-dependent manner, but this response was markedly suppressed in the presence of ABT-627 or EIPA [23]. Also, in the report by Isaka et al., exogenously applied ET-1 prolonged the duration of ventricular fibrillation in reperfusion in guinea pig hearts. Furthermore, ET-1-induced ventricular fibrillation was completely diminished by the combination of EIPA [24].

### 2.5. Exogenously Applied Big ET-1 in NE Overflow

It is well known that big ET-1 itself has no physiological effects independent of its conversion into ET-1 [42, 43]. Therefore, exogenously applied big ET-1 is thought to have qualitatively the same biological activities as ET-1. In our recent study using isolated perfused rat hearts, however, exogenous big ET-1 (0.1, 0.3 and 1 nM) did not increase NE overflow induced by 40-min global myocardial ischemia as opposed to the case of exogenously applied ET-1, in spite of the fact that ET-1 levels in coronary effluent from the heart exposed to protracted ischemia were dose-dependently increased by exogenous big ET-1 application [41]. To be more precise, a middle dose of this precursor peptide (0.3 nM) has a significant inhibitory action on NE overflow in ischemic hearts. This action was markedly attenuated by treatment with SM-19712 or A-192621, suggesting that exogenously applied big ET-1 is converted to ET-1 by ECE, locally in the heart, and this ET-1 binds to ET_B_Rs to exert its related beneficial action. However, it is important to note that the dose-response curve of the exogenous big ET-1-induced depressive effect on NE overflow was bellshaped (0.3 > 1 > 0.1 nM). One of the possible reasons is that ET_B_Rs may already be saturated by treatment with a middle dose of big ET-1, considering the result that combination of exogenous big ET-1 (0.3 nM) and ABT-627 did not produce a synergetic effect on NE overflow. Briefly, it has been proposed that a certain amount of ET-1 generated from exogenously applied big ET-1 preferentially acts on ET_B_Rs, but excessively generated ET-1 acts also on ET_A_Rs in ischemic hearts. We hypothesize functional coupling of ET_B_Rs and ECE-1, a dominant subtype of ECE, as for the reason why this phenomenon occurs. ECE-1 is classified into four isoforms based on differences in subcellular distribution [44]. Emoto et al. described that the isoform cleaving big ET-1 is different between endogenously generated and exogenously applied ET-1; the former is mainly catalyzed by isoforms expressed intracellularly, whereas the latter is cleaved by those expressed on the cell surface [45]. Based on this paper, endogenous ET-1 should be mostly generated inside the cell, whereas exogenously applied big ET-1 may be cleaved at the cell surface. In short, this isoform expressed on the cell surface would be positionally and/or functionally coupled to ET_B_Rs. This is just a hypothesis and must be further investigated.

As mentioned above, although ET_B_Rs have been demonstrated to exist in cardiac sympathetic nerves [24], it is unclear whether the effect seen at the middle dose (0.3 nM) of exogenous big ET-1 is through the activation of ET_B_Rs existing at this site. However, this may not be the case because the exogenous big ET-1-induced inhibitory effect on NE overflow after 40-min global ischemia/reperfusion was completely cancelled by treatment with the nitric oxide (NO) synthase (NOS) inhibitor N^G^-nitro-L-arginine (NOARG) [46]. This finding suggests the involvement of NO derived from NOS in ET_B_R-mediated inhibitory modulation of carrier-mediated NE release. Although there are three different isoforms of NOS, neuronal NOS (NOS1), inducible NOS (NOS2), and endothelial NOS (NOS3), none have been confirmed to be normally distributed in sympathetic nerves [47, 48]. Therefore, ET-1 generated from exogenously applied big ET-1 is expected to bind to ET_B_Rs existing in cells other than sympathetic nerves, for instance endothelium, cardiomyocyte, or nitrergic nerves. Further studies are required to clarify the role of ET_B_Rs existing in cardiac sympathetic nerves in carrier-mediated NE release.

There are a number of possible mechanisms for negative modulation of carrier-mediated NE release by NO. As noted above, carrier-mediated NE release from cardiac sympathetic nerve endings is triggered by inhibition of ATPase activity due to ATP depletion and activation of NHE [12]. In this regard, NO has been demonstrated to improve Na^+^/K^+^ ATPase function [49] or inhibit NHE function [50, 51] under cardiac pathological conditions. On the other hand, in culture experiments using PC-12 cells, another investigation has shown that NO negatively modifies NET activity in sympathetic neurons by nitrosation of regulatory sites on the transporter [52]. Again, further research is required to identify how NO suppresses carrier-mediated NE release from cardiac sympathetic nerve endings in protracted myocardial ischemia.

The inhibitory effect of exogenous big ET-1 on NE overflow (0.3 > 1 > 0.1 nM) correlated well with the recovery of left ventricular systolic and diastolic function after ischemia/reperfusion in isolated perfused rat hearts [41]. Briefly, big ET-1 at a concentration of 0.3 nM most efficiently improved left ventricular dysfunction after reperfusion. In addition, this beneficial effect of exogenously applied big ET-1 was canceled by respective treatment with SM-19712, A-192621, and NOARG [41, 46].

## 3. Clinical Implications

It is impossible to directly apply the above findings to a clinical setting mainly because of ex vivo data. However, previous in vivo studies of myocardial ischemia in animal models have provided clinically relevant information [25–29, 53]. In accordance with ex vivo data, each of the selective ET_A_R antagonists [26, 28], the nonselective ET_A_R/ET_B_R antagonist [25, 27, 28] and the ECE inhibitor [29] have been reported to suppress increases in NE levels resulting from myocardial ischemia. For example, Fraccarollo et al. [26] demonstrated that long-term treatment with the selective ET_A_R antagonist LU135252 decreased plasma NE concentrations 12 weeks after proximal left coronary artery ligation in rats. Lee et al. [28] showed the inhibitory effect of the selective ET_A_R antagonist ABT-627 or nonselective ET_A_R/ET_B_R antagonist bosentan on regional myocardial interstitial and left ventricle NE levels 4 weeks after myocardial infarction induced by ligation of the anterior descending artery in rats. In this study, these ETR antagonists improved left ventricular systolic and diastolic function in postinfarcted rats. Also, in a study by Mulder and associates [25], bosentan decreased plasma NE levels in rats subjected to permanent left coronary artery ligation for 2 or 9 months. Likewise, Kolettis et al. [27] reported that bosentan markedly decreased serum NE levels 24 h postligation of the left coronary artery as well as the total duration of ventricular tachyarrhythmias during the delayed phase (1–24 h) postligation in rats. In addition, an orally active ECE inhibitor PP36 has been indicated to suppress cardiac tissue NE release 48 h after acute myocardial ischemia due to microsphere embolization of coronary microcirculation in rats [29]. Left ventricular contractility was partially restored and distention significantly improved by PP36 treatment in this research. Furthermore, an in vivo study using a genetic model was also consistent with the results of an ex vivo study. Oikonomidis et al. [53] recently demonstrated that serum NE levels during the early phase of myocardial infarction induced by left coronary artery ligation were much higher in ET_B_R-deficient *sl*/*sl*  rats than in wild-type rats. In this study, the severity of ventricular tachyarrhythmias during the 1st h postmyocardial infarction was also greater in ET_B_R-deficient rats, indicating that ET_B_R decreases sympathetic activation and arrhythmogenesis during the early phase of myocardial infarction. Taken together, ET_A_R antagonists or ECE inhibitors can be expected to suppress sympathetic overactivity with excessive NE release and following cardiac dysfunction in patients with ischemic cardiac disease. As a matter of course, however, we must keep in mind that NE overflow is only one of the risk factors for the development of cardiac injury, and also that ET-1 possesses many other pathophysiological actions. In other words, although this paper focused on functional myocardial damage caused by ischemia, we should not forget that morphological alterations such as infarction, hypertrophy, apoptosis, or fibrosis are also contributive to myocardial ischemia/reperfusion injury.

## 4. Conclusion

**Figure 1:**
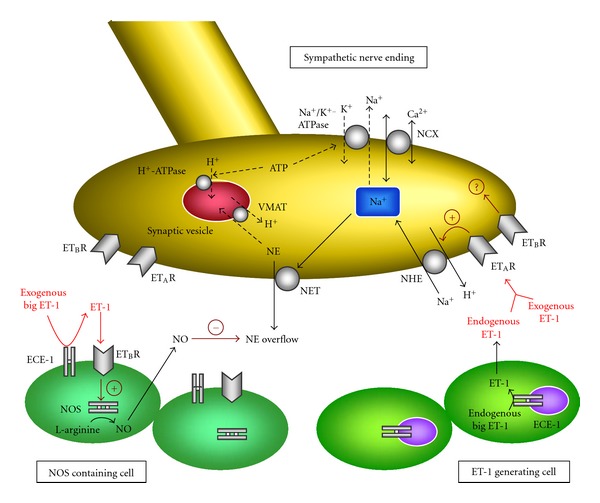
Summarizing scheme illustrating the interaction between the ET-1 system and carrier-mediated NE release in protracted myocardial ischemia. Stimulation of ET_A_Rs existing in sympathetic nerve endings by endogenously generated or exogenously applied ET-1 increases neuronal NHE activity, thus potentiating carrier-mediated NE release. In contrast, exogenously applied big ET-1 is converted to ET-1 by cell surface ECE-1, and this ET-1 preferentially binds to ET_B_Rs located on NOS containing cells to produce NO. Endogenously generated NO works to prevent carrier-mediated NE release. NCX, Na^+^/Ca^2+^ exchanger; VMAT: vesicular monoamine transporter.

In summary (see Figure 1), during protracted myocardial ischemia, endogenously generated or exogenously applied ET-1 activates ET_A_Rs in sympathetic nerve endings and stimulates neuronal NHE. As a result of NHE stimulation, NE is excessively released by potentiating carrier-mediated efflux. In contrast, ET-1 produced by appropriate amounts of exogenous big ET-1 works to prevent NE overflow by preferentially binding to ET_B_Rs located on NOS containing cells and producing NO. On the other hand, the role of ET_B_R at cardiac sympathetic nerves in carrier-mediated NE release remains to be determined. Finally, a better understanding of the ET-1 system in NE overflow induced by protracted myocardial ischemia can lead to better treatments for ischemic cardiovascular diseases.
